# Women’s self-rated attraction to male faces does not correspond with physiological arousal

**DOI:** 10.1038/s41598-017-13812-3

**Published:** 2017-10-19

**Authors:** S. Hagerman, Z. Woolard, K. Anderson, B. W. Tatler, F. R Moore

**Affiliations:** 1 School of Social Sciences, University of Dundee, Dundee, Scotland UK; 2 School of Psychology, University of Aberdeen, Aberdeen, Scotland UK

## Abstract

There has been little work to determine whether attractiveness ratings of faces correspond to sexual or more general attraction. We tested whether a measure of women’s physiological arousal (pupil diameter change) was correlated with ratings of men’s facial attractiveness. In Study 1, women rated the faces of men for whom we also measured salivary testosterone. They rated each face for attractiveness, and for desirability for friendship and long- and short-term romantic relationships. Pupil diameter change was not related to subjective ratings of attractiveness, but was positively correlated with the men’s testosterone. In Study 2 we compared women’s pupil diameter change in response to the faces of men with high versus low testosterone, as well as in response to non-facial images pre-rated as either sexually arousing or threatening. Pupil dilation was not affected by testosterone, and increased relatively more in response to sexually arousing than threatening images. We conclude that self-rated preferences may not provide a straightforward and direct assessment of sexual attraction. We argue that future work should identify the constructs that are tapped via attractiveness ratings of faces, and support the development of methodology which assesses objective sexual attraction.

## Introduction

Men’s faces hold cues to levels of circulating and pubertal testosterone. Masculine male facial features, such as a wide jaw and heavy brow ridge, develop under the action of the pubertal surge in testosterone (T)^1^, and there are correlations between salivary T and facial masculinity in adulthood^2,3^. The role of T in facial attractiveness, however, is complex. Studies have variously shown women’s preferences for male facial cues to high T, preferences for facial cues to low T, and no preference (see^4^ for a review). This has been attributed to methodological differences between studies^4,5^, but is also likely to be due to the multiple physiological and behavioural consequences of T which women must trade-off when judging the desirability of potential partners.

T is associated with aggression^6^, dominance^7^, and hostility^8^, and masculine male faces are perceived as more dominant and less likely to make a suitable partner^9^. Therefore cues to T in the face are likely to contribute to negative judgements of personality and attractiveness. As, however, T may also act as an immunosuppressant^10^, masculine male faces may provide an honest indication of the strength of the bearer’s immune system^11–13^. This, conversely, should result in positive attractiveness judgements of masculine-faced men. Given the multiple traits signalled by cues to T in the face, it is perhaps unsurprising that women’s judgements of their attractiveness are facultative. Women, for example, may prefer cues to high T during the fertile phase of the menstrual cycle when genetically determined immunocompetence can contribute to the health of any resultant children^14,15^, in the context of a short-term relationship, when personality traits are less important^16^, or when living in an area with poor health outcomes^17^. In addition, there may be individual differences in the extent to which cues to T are considered attractive with, for example, women who perceive themselves as having a higher mate value showing stronger preferences for masculine faces^18^.

Research into face preferences, however, has been almost exclusively limited to ratings of the attractiveness of faces. Most research asks participants to simply indicate ‘How attractive is this face?’ How this is interpreted by the participant remains unclear. We do not know whether participants are rating faces on the basis of sexual attraction, more general interpersonal attraction (e.g. treating ‘attractiveness’ as ‘friendliness’), or some other construct. Exceptions to this have included comparison of self-reported preferences for faces with gaze fixation on images that differ in masculinity. Results, however, have been contradictory with^19^ showing that women attended more to masculine male faces, and^20^ showing the opposite. One possibility is differences in the fixations included in analyses (first fixation in the former, and total fixation count and duration in the latter) and inclusion of male participants in one sample^20^. Here, then, we sought to determine (a) whether rated attractiveness was correlated with an objective measure of arousal (pupil diameter change), and (b) whether cues to T in men’s faces were correlated with either rated attractiveness or objective measures of arousal. Furthermore, we sought to clarify whether any pupil diameter changes in response to cues to T in men’s faces were due to sexual arousal or more general arousal.

In Study 1 we measured changes in women’s pupil diameter in response to images of the faces of men that differed in levels of circulating T, and asked them to rate the faces for attractiveness and desirability for short- and long-term romantic relationships, and for friendship. Pupillary responses occur primarily due to the light reflex (constriction and dilation that happens during changes in light) or the accommodation reflex (constriction and dilation when focusing on an object^21^). Smaller changes in pupil diameter, however, indicate cognitive processing and are treated as a measure of arousal^22^. Hamel^23^ found that subjective reports of sexual arousal were correlated with the pupillary reactions of female students when watching images of male models. As there was no such relationship when viewing same-sex images, the authors concluded that changes in pupil diameter were a proxy of sexual arousal. Pupil dilation was similarly found to discriminate between sexual arousal (i.e. in response to viewing an erotic movie) versus more general arousal (i.e. in response to watching a suspense movie) in men^24^. More recently^25^, reported an increase in women’s mean pupil diameter during their fertile menstrual cycle phase in response to sexual stimuli of their current partner. While, then, there is evidence to support using pupil dilation as a measure of sexual arousal, we sought to control for the possibility that any pupil diameter change in response to cues to T in men’s faces were due to more general arousal (e.g. high T male faces may be perceived as more threatening). In Study 2 we compared women’s pupil dilation in response to the faces of men with high and low T, with responses to non-facial stimuli that were sexually arousing or threatening.

## Study 1

### Results

Table 1 above shows correlation coefficients for relationships between all variables included in analyses, and means and standard deviations. When ratings of desirability for long- and short-term relationships, attractiveness and desirability for friendship were entered into a factor analysis, a single factor was extracted with an eigenvalue of 3.46 and which accounted for 86.44% of the variance. Factor loadings were 0.89 for ratings of desirability for friendship, 0.95 for desirability for a long-term relationship, 0.93 for desirability for a short-term relationship, and 0.95 for attractiveness.

**Table 1:** Spearman’s correlation coefficients for relationships between all variables included in analyses, and means and standard deviations.

	Correlations					
	Mean (SD)	Pupil diameter change (%)	Desirability for friendship	Attractiveness	Desirability for long-term relationship	Desirability for short -term relationship
Testosterone (ng/mL)	0.30 (0.11)	0.36*	−0.14	−0.14	−0.16	−0.06
Desirability for short-term relationship	1.79 (0.38)	0.21	0.76**	0.80**	0.88**	—
Desirability for long-term relationship	2.11 (0.40)	0.09	0.80**	0.85**	—	—
Attractiveness	2.25 (0.58)	0.17	0.82**	—	—	—
Desirability for friendship	2.48 (0.51)	0.34*	—	—	—	—
Pupil diameter change (%)	2.42 (2.93)	—	—	—	—	—

*p < 0.05, **p < 0.001.

**Figure 1:**
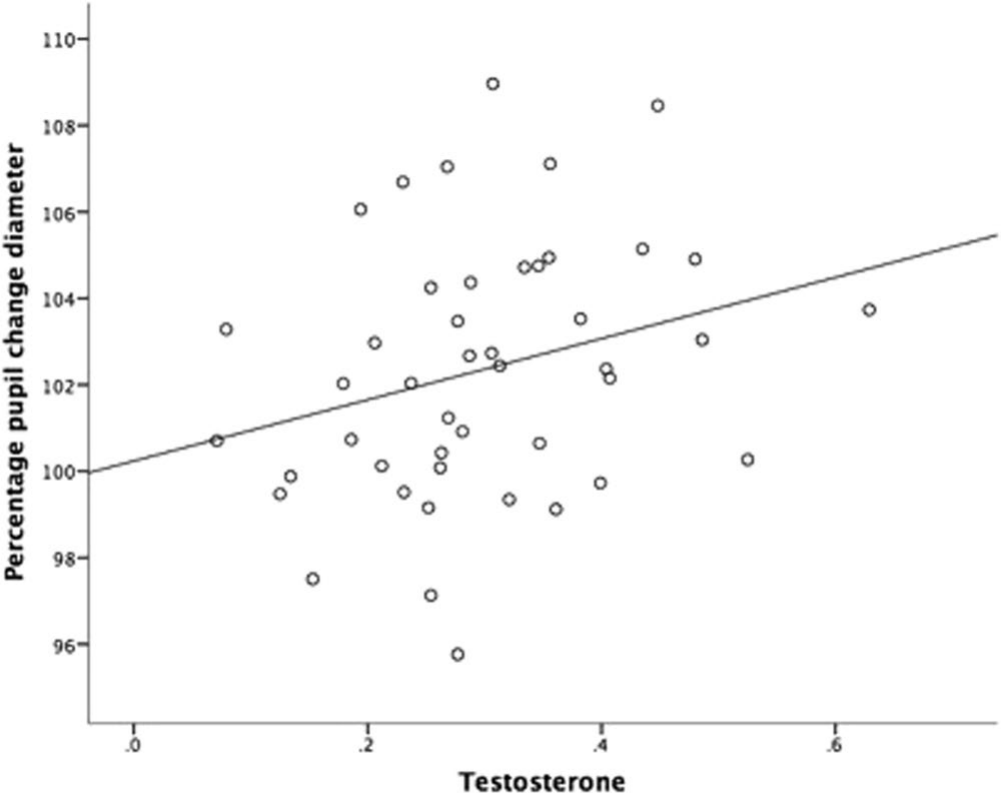
Relationship between salivary testosterone (ng/mL) of men and the average percentage change in pupil diameter of women when viewing the men’s faces.

When the ratings factor and T were entered as predictors in a linear mixed effects model to predict pupil diameter change, we found that change in pupil dilation was predicted by T, β = 9.52, SE = 3.63, t = 2.63, p = 0.012 (see Fig. 1). The ratings factor approached significance, β = 0.83, SE = 0.43, t = 1.93, p = 0.0611. Higher T in the face and higher positive ratings were associated with a greater increase in dilation of the pupil (relative to the baseline pupil dilation).

### Discussion

Our results show that women’s pupil diameter increased in response to faces of men with high T. They also increased in response to faces which had been rated as desirable for long- and short-term relationships and friendship, and attractive, although this effect was only marginally significant. Therefore, our results show that women experience greater arousal when viewing the faces of men with high levels of T.

In zero-order correlations, attractiveness ratings were significantly positively correlated with desirability for all 3 relationship types (long- and short-term romantic relationships, and friendship). This suggests a halo effect, in which men who are rated as ‘attractive’ (e.g. who may have a healthy appearance) are attributed with positive ratings on all dimensions.

In zero-order correlations we did not find significant relationships between women’s pupil diameter change and their ratings of the attractiveness, or desirability for short- or long-term romantic relationships of men’s faces. There was, however, a significant relationship between pupil diameter change and desirability for a friendship. There was also a significant relationship between pupil diameter change and T. These results appear to be in opposition to one another. Pupil dilation in response to the faces of men perceived as desirable for friendship seem unlikely to be due to sexual arousal, particularly since pupil diameter did not correlate with preferences for faces perceived as a attractive or desirable for a short-term relationship. One possibility is that pupil dilation occurs as the result of arousal in general, rather than sexual arousal. For example, pupil dilation in response to the faces of men with high T may be due to perceiving them as sexually arousing or threatening. In Study 2 we compared pupil diameter changes in response to faces of men with high and low T with those in response to non-facial stimuli that were either sexually arousing or threatening.

## Study 2

### Results

**Figure 2:**
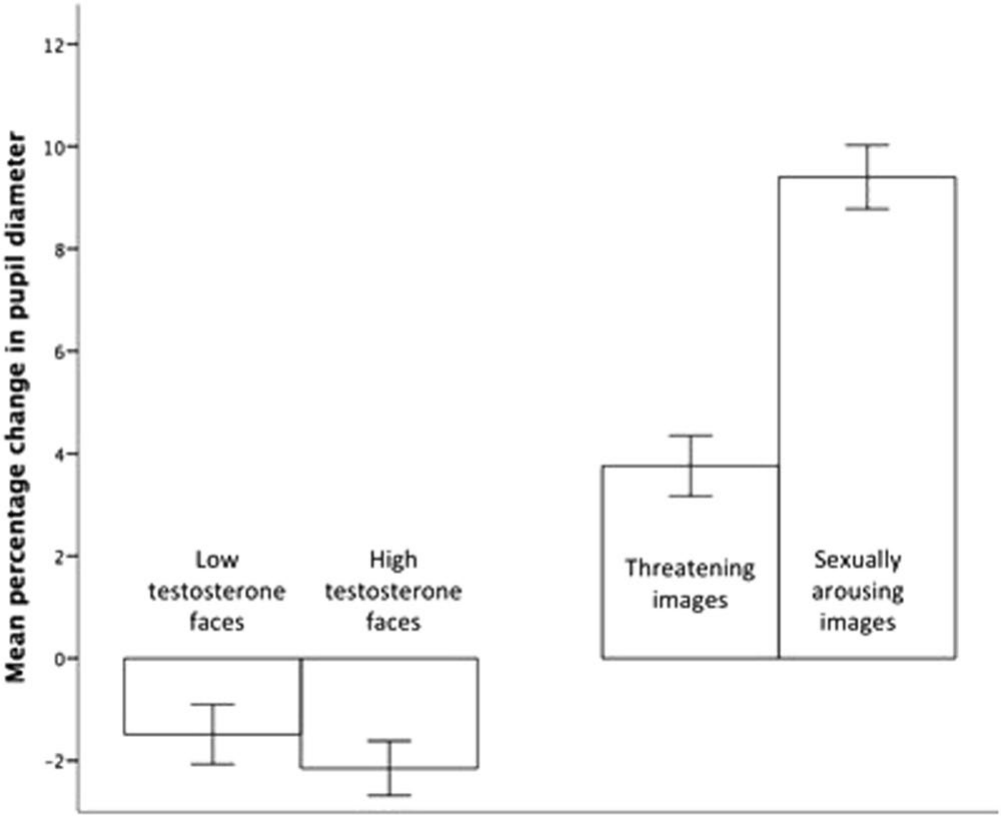
Mean percentage change in pupil diameter of women viewing faces of men with low and high testosterone, and when viewing threatening and sexually arousing images (+- 1 standard error).

Table 2 above shows the pupil diameter change for each condition. For faces, there was no difference in pupil diameter change between low T and high T faces, β = 0.22, SE = 0.83, t = 0.27, p = 0.79. For scenes, there was a greater increase in pupil diameter when viewing sexually arousing images than when viewing threatening images, β = 5.56, SE = 0.82, t = 6.75, p < 0.001. See Fig. 2.

**Table 2:** Mean (and standard deviation) pupil diameter change (% pixels) across 4 stimuli conditions.

	High T faces	Low T faces	Sexually arousing images	Threatening images
Change in pupil diameter (%)	−1.39 (11.8)	−1.16 (12.6)	10.1 (12.8)	4.46 (12.4)

### Discussion

The results of Study 2 fail to replicate our finding that high T faces are associated with an increase in pupil diameter. Furthermore, although increases in pupil diameter were greater for sexually arousing than threatening stimuli, pupil diameter increased in response to both sets of stimuli, so it is difficult to conclude whether the positive association between T and pupil diameter change in Study 1 was due to sexual arousal in response to the faces of men with high T, perceived threat, or a combination of the two.

## General Discussion

We set out to answer 3 questions: (1) do attractiveness ratings of men’s faces by women correspond with ratings of desirability for a long- or short-term romantic partner or for a friendship? (2) are ratings correlated with pupil diameter change? and (3) how do ratings and pupil diameter change correlate with the men’s T levels?

Attractiveness ratings were correlated with desirability for all 3 relationship types, suggesting that attractiveness ratings tap into a more general positive ‘liking’ than sexual attraction, or attraction in the context of a romantic relationship, specifically. Alternatively, women may prefer attractive male friends for the purpose of extra-pair sexual relationships or future romantic relationships. This was supported by the failure to detect relationships between ratings of attractiveness and desirability for a romantic relationship with pupil diameter change, suggesting that these self-reported preferences do not provide a reliable assessment of attraction. Ratings of desirability for a friendship, however, were correlated with pupil diameter change, suggesting greater arousal when finding a male face desirable for friendship. One possibility is that the men that women consider desirable as a friend are those who are perceived as being dominant and aggressive and, therefore, able to offer support and protection. T and desirability for friendship ratings, however, were not correlated in this sample.

Women experienced greater physiological arousal in response to the faces of men with high T than those with low T in Study 1 but not in Study 2. It is difficult to determine why we found significant associations in one study and not the other. It is possible that the comparison of groups of faces in Study 2 (2 sets of 10 faces that were either the highest or lowest T in the sample) obscured the association between T and pupil diameter change that was detected using correlational analyses across the whole sample of faces in Study 1. Our attempts to interpret the significant association reported in Study 1, however, should be taken in the context of the failure to replicate the finding in Study 2. We speculated that the greater pupil dilation in response to high T male faces could be due to either sexual arousal or perceived threat associated with masculine faces. In Study 2 we found that pupil diameter increased in response to both threatening and sexually arousing non-facial stimuli, but significantly more so for the sexually arousing images. Again, however, we are cautious in interpreting this finding as support for pupil dilation in response to high T faces being due to greater sexual attraction to these faces since pupils also dilated in response to threatening images. We suggest that future work should seek to address a limitation of our work in that faces were only masked for perceptual ratings, but masked and luminance controlled for pupil dilation measurements. In follow-up work, both tasks would employ the same controls. It would be interesting to explore the relationship between T, and pupil dilations with further perceptual measures such as masculinity and threat.

Overall, then, our results suggest that the faces that women rate as attractive or desirable for a relationship are not those which provoke the greatest physiological arousal. We argue that future work should seek to better understand the constructs of judgement assessed by attractiveness ratings of faces, and to disentangle perceptions of men’s faces on the basis of cues to T.

## Methods

Both studies were approved by the University of Dundee Research Ethics Committee (reference number UREC 15055), and all methods were performed in accordance with the relevant guidelines and regulations. All participants provided informed consent.

## Study 1

### Face-rating task

#### Participants

Sixty-nine heterosexual, causcasian, women (age: 27.72 (10.13)) were recruited via convenience sampling on social media.

#### Materials

Forty-seven Caucasian male faces were used for the rating tasks. The methods of image collection and assessment of circulating testosterone from saliva samples are described in^26^. Participants were asked to rate faces on 1–7 likert scales for one of the following: attractiveness (22 raters; Cronbach’s alpha = 0.93), friendliness (11 raters; Cronbach’s alpha = 0.97), desirability for a short-term relationship (21 raters; Cronbach’s alpha = 0.96), and desirability for a long-term relationship (15 raters; Cronbach’s alpha = 0.99).

#### Procedure

Participants were directed to the face-rating task by clicking on a web-link. They were allocated to their rating task via a random allocation script. After providing basic demographic information, participants rated the faces, which were masked and presented in random order.

### Pupil Dilation

#### Participants

Twenty female students were recruited from the University of Dundee (age: 22.25 (1.75)).

#### Materials

The 47 male faces described above were used as stimuli in a pupil diameter change task. Faces were prepared for display by masking to obscure hair and clothing, cropping to a standard size (for this, faces were aligned and normalized on inter-pupillary distance), set to grayscale, and setting against a black background. For each face stimulus, we created an image containing a block of uniform intensity pixels that was matched for size and that contained the mean luminance of the facial stimulus, using Matlab. This allowed us to control for any pupil diameter change that was due to differences in luminance between faces. The laboratory used for testing had no natural light so that luminance in the room was standardised.

#### Eye tracking

Pupil dilation was measured with an Eyelink 1000 eye-tracker, sampling pupil size in the participant’s dominant eye at 1000 Hz. Data were gathered for the participant’s dominant eye. Any saccades were detected with the SR Research algorithm using default sensitivity settings. Pupil size estimates from the eye tracker were discarded during any identified saccades or blinks, such that data analysed and reported here are only for samples occurring during steady fixation.

#### Procedure

Participants completed a brief questionnaire to assess demographic information, and ocular dominance was assessed via the Porta test. Here the participant was asked to extend one arm and, with both eyes open, to align their index finger with a distant object. They then alternated closing each eye to determine which eye is viewing the object (i.e. the dominant eye). Participants placed their chin in a height adjustable headrest in order to reduce head movements. They were then asked to look at the screen while the researcher adjusted the camera to attain adequate resolution of the pupil. Calibration was conducted by recording the eye position at 9 standard calibration points, appearing as black dots with a white middle on a white background, corresponding to a regularly spaced 3 × 3 matrix. A second 9-point array of dots was used to obtain an estimate of the calibration accuracy. Calibration – and if necessary eye tracker setup – was repeated until the validation showed spatial accuracy of gaze estimation to be better than 0.5 degrees on average and no worse than 1 degree for any of the 9 calibration points. Eye position is important for the precise computation of pupillary horizontal and vertical diameters, as the projection of the eye pupil in the camera changes with eye position. Finally, participants were instructed to fixate on the screen and maintain their gaze at all times on the slideshow of images that would appear. The order or the images was randomised for each subject and pupillary diameters were recorded throughout the viewing of the images, starting from the onset of the picture on screen and for the duration of 10 seconds. Before each face was displayed, the corresponding color block stimuli was displayed for 10 seconds.

### Analysis

We first confirmed linearity of relationships by graphing and inspecting the data. We then explored the correlations between T, the ratings collected for each face, and the change in pupil diameter from baseline recorded in the eye tracking phase of the study. These correlations are between average ratings and pupil diameter change for each face. We then ran a linear mixed effects model (LMM) to predict change in pupil diameter from baseline on each face with T, and a factor combining ratings of desirability for short-term and long-term relationships, attractiveness, and desirability for friendship, as fixed effects. Participant and item (face exemplar) were entered as random effects, each with intercept and random slopes calculated for each fixed effect (a maximal model, as recommended by^27^). LMMs were run using the lmer() function of the lme4 package^28^ in the R programming environment^29^. For each model, we report the predictors’ coefficients (β-values), the SE-values, t- values, and the associated p-values generated using the lmerTest library^30^.

## Study 2

### Participants

Forty female students were recruited from the University of Dundee (mean age = 22.98 (4.47)).

### Materials

A set of 20 male faces were selected from the those described for Study 1: the 10 with the highest T levels and 10 with the lowest T levels. From these, we removed each face with facial hair and replaced it with the face of the next highest (or lowest) T man. The groups differed significantly in T (t_18_ = −7.39, p < 0.001), but not in attractiveness, health, age, or masculinity (all p > 0.12). Faces were masked, cropped, and set against a black background.

We selected 20 images from the International Affective Picture System^31^. This is a dataset of normative, standardized, emotionally-evocative, colour photographs. All photographs have been pre-rated on a number of characteristics. We sorted them on the basis of ‘pleasantness’ ratings made by women (these ratings are provided with the database) and selected 15 images from below the median which had the highest female arousal ratings and contained images of guns, knives, or attacks (i.e. *threatening* condition), and 15 from above the median with the highest female arousal ratings and which contained erotic scenes (i.e. *sexually arousing* condition). There was a significant difference for arousal with the erotic images (mean = 6.81, SD = 0.51) being more arousing than the violent images (mean = 1.97, SD 0.17) (t(18) = −28.92, p < 0.001), so we removed the 5 images with the highest arousal ratings from the *sexually arousing* condition, and the 5 images with the lowest arousal were removed from the *threatening* condition (*sexually arousing*: mean = 1.46 (0.22); *threatening*: mean = 1.49 (0.21); p = 0.76).

### Procedure

After completing a short questionnaire of demographic information, carrying out the Porta test and eye-tracker calibration, participants viewed the images following the methodology described for Study 1, with the exception that the images shown during the baseline 10-s viewing period were scrambled versions of the experimental stimuli, such that each pixel within the face or scene image was shown but in a random position. In this way the control patch had the same mean luminance but also the same distribution and range of brightnesses as the experimental image. These scrambled images were also shown against a black background.

### Analysis

Linear mixed effects models were separately for faces and scenes, with T (low, high) and scene content (sexually arousing, threatening) as dummy coded categorical fixed effects. Participant and item (face exemplar) were entered as random effects with random slopes and intercepts calculated for the fixed effect in both models.

### Data Availability Statement

The datasets generated during and/or analysed during the current study are available from the corresponding author on reasonable request.

## Electronic supplementary material

Dataset for Study 1

Dataset for Study 2
